# Acute General Peritonitis from Perforation of Carcinoma Coli

**Published:** 1907-09-14

**Authors:** 


### Illustrative Cases.

### ACUTE GENERAL PERITONITIS FROM PERFORATION OF CARCINOMA COLI.

The two following cases illustrate the fact that, in operating for acute general peritonitis, it is not at all easy to be sure beforehand what is going to be found. Carcinoma of the colon is amongst the possibilities, though it is certainly not so common a cause of suppurative peritonitis as are appendicitis or perforated gastric or duodenal ulcer. Growth in the colon most often causes one or other of two kinds of symptoms, namely: (1) Those of acute intestinal obstruction. (2) Those of secondary deposits, particularly in the liver, with jaundice, or ascites, and cachexia.

Some cases, however, first come under observation on account of symptoms of general peritonitis of acute onset. Either the growth itself becomes sloughy and ulcerated until there is a hole through it into the peritoneal cavity, or else there is so much distension of the bowel either immediately above the growth or else in the cæcum, that stercoral ulceration arises, and is followed by perforation. The peritonitis manifestly indicates the necessity for immediate laparotomy. It will be more than likely that appendicitis will be the provisional diagnosis beforehand; if, however, this is found not to be the case when the abdomen has been opened, and some other lesion has to be sought for, it is well to put the hand down into the left iliac fossa and pelvis to see if there is not a growth of the sigmoid colon, particularly if the patient is over 50, and the history does not point directly to trouble in the stomach or duodenum; in a woman the pelvic viscera can be examined at the same time in case there should be a pyosalpinx or an ovarian cyst with twisted pedicle.

Case 1.—A man, aged 51, had been unable to work for eleven years on account of chronic osteo-arthritis, but had otherwise been in excellent general health. At 6 p.m. one evening he complained suddenly of acute pain in the lower part of his abdomen, he vomited a little, retched considerably, and had great difficulty in defæcation. A small motion was passed. He did not call in a doctor, and next morning he was a little easier. That night, however, he had another attack of general abdominal pain accompanied by vomiting; he took castor oil, after which the bowels were very freely opened, and yet the pain continued as badly as before, or worse. He sent for a doctor next morning; that is to say, when 38 hours had elapsed from his first attack of pain. The abdomen was now considerably distended, moderately rigid all over, but still moving fairly on respiration. There was much more tenderness below the umbilicus than above it, though the pain was general with its maximum at the navel. The bladder was distended up to three inches above the pubes; there was an impaired note in either flank, not shifting with change in the patient's position; and there was a friction-rub to be heard over the spleen. This rub was most helpful in deciding that there was general peritonitis. The patient's tongue was parched and brown; the pulse rate was 120, the respiration rate 28, the temperature 98° F. The lungs, heart, and the urine, drawn off by catheter, were natural. Rectal examination was negative. A median incision was made from umbilicus to pubes, and a considerable amount of mud-coloured fluid welled up from the peritoneal cavity. The intestines were distended, crimson, and covered with patches of lymph. The vermiform appendix was found to be no more inflamed than the rest of the bowel. There were many recent adhesions between the coils; on further examination a hard inflamed mass was found in the sigmoid flexure, some three or four inches above the rectum, and outside the mass was a local collection of pus. The patient's condition was very bad, so that the operation had to be hurried; the gut above the mass was incised and a Paul's tube tied in; the peritoneal cavity was drained, and the patient put back to bed. For a week after the operation he continued to improve, and with a little more good fortune he would have survived. After a week, however, he began to sink, and died on the thirteenth day after the original operation, At the autopsy the carcinoma was found to be of the soft fungating ulcerated type, five inches long, and nearly as big as a fist. There was little or no stenosis of the lumen of the bowel; the growth was beginning to infiltrate the mesosigmoid, but there were no secondary deposits either in the lymphatic glands or in the liver. Had the patient recovered from the effects of the general peritonitis, excision of the growth should have cured him completely.

Case 2.—A man, aged 56, had always been constipated, and five years ago he developed an inguinal hernia, for which he wore a truss. Three months ago he had a sudden pain in the lower part of his abdomen, lasting a few minutes only; since then he had had several such attacks. The attacks were very short, and unaccompanied by vomiting. Except for chronic constipation he made no complaint about his bowels; the constipation was no worse than it had been for a long time past; rectal examination was negative; the heart, lungs, and other viscera seemed natural. The diagnosis was obscure, though appendicitis, with adhesions, seemed probable. One day he was suddenly seized with acute abdominal pain vomiting, and prostration, with all the typical signs of acute general peritonitis. Laparotomy was performed, and a large abscess in the appendicular region was discovered and drained, whilst the general peritonitis was also found and dealt with. The patient lived three days longer, but died exhausted. At the autopsy the perityphlitic abscess was found not to be of appendicular origin, but due to stercoral ulceration and perforation of the cæcum, arising from obstruction to the colon by an annular carcinoma of the colon three feet above the anus. The cause of the general peritonitis was not the perityphlitic abscess, which was well shut off by adhesions, but a second perforation of the colon immediately above the stenosing growth. There were numerous secondary deposits in the abdominal lymphatic glands near the descending colon. The real cause of the acute general peritonitis was perforation in connection with the unsuspected carcinoma coli.

